# Assessing Factors That Influence Womenpreneurs’ Intention to Use Technology: A Structural Equation Modeling Approach

**DOI:** 10.3390/bs13020094

**Published:** 2023-01-23

**Authors:** Yung-Tsan Jou, Wei-Jung Shiang, Riana Magdalena Silitonga, Muna Adilah, Audrey Zebe Agathon Halim

**Affiliations:** 1 Department of Industrial and Systems Engineering, Chung Yuan Christian University, Taoyuan City 320314, Taiwan; 2 Department of Industrial Engineering, Atma Jaya Catholic University of Indonesia, Jakarta 12930, Indonesia

**Keywords:** empirical study, financial literacy, technology adoption, womenpreneurs

## Abstract

The growth of financial literacy is significant nowadays. Because of this, more people are becoming increasingly responsible in their financial planning, investments, and living expenses. In developing new technology, it is necessary to know the technological acceptance of the prospective users of the technology itself. This study aims to identify the primary factors influencing the technology acceptance levels of lower-middle socio-economic users for a digital financial literacy application. The proposed model in this research was developed based on UTAUT, TAM, and Usability model, and it consists of six primary constructs: (1) Performance Expectancy; (2) Effort Expectancy; (3) Social Influence; (4) Resources and Cost; (5) Satisfaction; (6) Behavior Intention. All the hypotheses used in this study were statistically measured using SmartPLS tools. This study found that because many lower-middle socio-economic users lack sufficient understanding of technology to properly utilize it, that a digital platform is not the right tool to teach them financial literacy.

## 1. Introduction

The growth of financial technology and literacy are significant nowadays. Because of this, more people are becoming increasingly responsible in their financial planning, investments, and household budgeting. Financial literacy affects every aspect of financial decision making and has implications for both individuals and society [1]. Financial literacy has two dimensions: basic financial knowledge and the ability to implement that knowledge [2]. Financial literacy positively influences decision-making outcomes. An individual who understands financial problems such as risk, rate of return, credit-card payment structure, and household budgeting tends to make better financial decisions [3].

The COVID-19 pandemic has had a significant impact on all sectors. Due to its rapid spread, governments worldwide issued regulations not to gather and to stay at home. Activities usually carried out face-to-face now had to be carried out online. Suddenly there was a change around the world that required people to be involved in e-learning and online activities to finish their work. While working from home became a solution to prevent the spreading of the virus during the pandemic, stay-at-home regulations also negatively impacted economies and individuals globally. Many people encountered great difficulty in earning a stable income during the pandemic era due to restrictions on activities outside the home. It made lot of people struggle to meet their basic living needs such as food and health, especially in developing nations [4].

According to the financial problems of societies during the pandemic, financial literacy is needed and should be applied in society, especially for women. Women are crucial to the world’s economy. Moreover, the percentage of women in the workforce in Indonesia expanded by 23% in 1996 and 33% in 2013. Unfortunately, women’s salaries remain 18% lower than men’s salaries. The statistics on reserve funds, investments, and loan levels all display a noteworthy gender gap. Once an effective program has been created to assist women in overcoming the challenges they confront, they will certainly receive more compensation and save more. Financially empowered women have a positive effect on the economy and help to create superior standards of living [5]. 

In Indonesia, statistical data [6] shows that as many as 64.5% of MSMEs in Indonesia are managed by women. This demonstrates women have high potential and passion for organizing and collaborating in cooperatives. High enthusiasm and great support among women’s cooperatives mean the number of new Micro Small Medium Enterprises (MSMEs) they establish keeps growing [7]. The position and role of women in the household does not dampen their enthusiasm for entrepreneurship. The time constraints of managing both their household and a business cause many women to choose businesses such as traditional retail stalls, the culinary arts, and fashion enterprises as options for entrepreneurship. Such businesses allow women to simultaneously manage the business they own and their domestic affairs [8].

The contribution of MSMEs to Indonesia’s economy now approaches 60% of the Gross Domestic Product (GDP). This contribution cannot be isolated from self-employed women, whose numbers in 2018 reached 14.3 million, and who contribute 9.1% to the Indonesia’s GDP [9].

However, even with such an incredible contribution, MSMEs still routinely face many obstacles, such as obtaining access to funding from financial institutions [10]. Current information shows that interaction levels of MSMEs with financial institutions is still very low, i.e., presently only 30% of them can access financing. These limitations on access to capital from financial institutions are a result of MSME’s having low or indeed non-bankable profiles, leading to their profit viewpoint being disregarded [11]. This is often due to the difficulty MSMEs experience in tracking their money, especially in arranging and managing their income and expenditure levels. Meanwhile, financial institutions require financial statements which reflect the current condition of MSMEs to support their applications for funding [12].

Based on the National Survey of Financial Literacy and Inclusion [13] report, the level of financial literacy in Indonesia remains low, at approximately 29.7%. It can be said that Indonesians lack adequate knowledge of and trust in financial institutions. Therefore, a financial literacy program is necessary to improve Indonesian society. The government, together with a leading Indonesian Islamic financial inclusion bank that focuses on women’s empowerment [14], has already initiated a strategy for providing financial literacy education to the people of Indonesia. Nevertheless, the pandemic affected these activities. Due to the COVID-19 outbreak, this teaching strategy was disrupted and eventually halted. Therefore, new innovations and technology are necessary to be able to provide financial literacy education. This study will examine the potential for acceptance of a financial literacy smartphone application currently under development.

When developing new technology, it is necessary to know the technological acceptance of the prospective users of the technology itself. The prototype e-learning platform will be tested by utilizing the Unified Theory of Acceptance and Use of Technology (UTAUT), Technology Acceptance Model (TAM), and Usability models, which are the most frequently used models that measure potential factors influencing acceptance [15]. 

The use of the UTAT model was already carried out by Gunawan et al. (2019) [16]. Their research identified five tested hypotheses: (1) Performance Expectancy has a positive impact on Behavioral Intention (BI); (2) Effort Expectancy on BI; (3) Social Influence on BI; (4) Facilitating on BI; and (5) Anxiety on BI. All the hypotheses in this study were declared “accepted”. The data also show that by using e-money in their business organizations can increase their performance gains. 

In addition, research conducted by Pal and Vanijja (2022) [17] analyzed the perceived Usability factor of Microsoft Teams using the modified System Usability Scale (SUS) and Technology Acceptance Model (TAM). The results showed similarities and equivalence between the two methodologies, with the construct of perceived ease of use of TAM having a more significant similarity with SUS. This study also considers whether the digital platform (mobile or web) affects perceived usability. The results show that the platform used (mobile or web) does not affect usability. In this study, the TAM variables used are perceived usefulness and ease of use.

Moreover, research was conducted by Sukendro et al. [18] on 974 students from five universities in Indonesia by implementing the extended Technology Acceptance Model (TAM) with the condition of the facility as an external factor. The results of this study demonstrate the proposed TAM-based scale has succeeded in explaining the factors that predict the use of e-learning among Indonesian sports and science students during the pandemic. It discovered that a significant relationship exists between facility condition and perceived ease of use, and between facility condition and perceived benefits. Additionally, there is a significant relationship between all of the core components of TAM except for the relationship between perceived benefits and attitudes. In that study, the TAM variables used are perceived usefulness, perceived ease of use, attitude, and behavioral intention.

Based on previous studies, this study will evaluate the prototype financial literacy smartphone application under development based on UTAUT, TAM, and Usability on technology acceptance of Micro Small Medium Enterprises (MSME) in Indonesia. The variables used are performance expectancy, effort expectancy, efficiency, resources and costs, satisfaction, behavior intention, and acceptance prototype.

This study aims to identify the primary factors influencing the technology acceptance of lower-middle socio-economic users for e-learning, specifically examining the prototype financial literacy smartphone application under development. This study also proposed an acceptance model adopted from UTAUT, TAM, and Usability.

## 2. Materials and Methods

Financial literacy is a component of human capital that incorporates insight and/or information, abilities, attitudes, and certainty related to monetary decision-making. The main operational variables in financial literacy are budgeting, saving, investing, and loaning [19]. In addition, financial literacy has two dimensions: knowledge of basic financial concepts and the ability to adopt the knowledge [2]. Financial literacy positively influences the decision-making process. Someone who understands more about financial issues such as risk, level of returns, credit card payment structures, and household budgeting tends to make better decisions [3]. Financial literacy influences all day-to-day and long-term financial decisions and has implications for individuals and society [1].

Research conducted by Morgan and Long [20] shows that financial literacy positively affects savings rates. People with a high level of financial literacy generally have both more formal and informal savings when compared to people with low levels of financial literacy. Financial literacy can also increase a worker’s confidence and productivity, improve workability, and help workers avoid financial difficulties [21].

The factors that affect the level of financial literacy are very diverse. Financial education has a significant effect on financial behavior and financial literacy level. In addition, income level also affects financial behavior and levels of financial literacy [22]. Research by Kaiser and Menkhoff and Anshika et al. (2021) [23] on MSMEs in India shows that the factor which most influences the financial literacy of MSME actors is the level of gross income from a business. The higher the gross income level of the business, the higher the owner’s level of financial literacy [23]. 

Research from The Organization for Economic Cooperation and Development (OECD) shows that the gender gap affects levels of financial literacy. Men have a higher score compared to women in financial literacy assessments, and there were more top-performing men than women. Moreover, women receive less education related to financial subjects compared with men whose high intentions include having good digital financial interactions, since this is likely to influence all aspects of life, thereby increasing the powerlessness of women as a whole. Therefore, enhancing the knowledge of financial literacy is essential, especially for women [24]. In addition, women have less spare time to study more about financial literacy because they are kept busy with domestic activities. Hence, using technology in adopting and teaching financial literacy is necessary. Before implementing the technology into financial literacy, the researchers need to know the probability of acceptance for that technology within the communities and or organizations that will use it. This aspect must be well considered to support future applications. Technology Acceptance Model (TAM) is one of the proposed models that can be used in this research.

Fred D. Davis introduced the term Technology Acceptance Model (TAM) in 1984. TAM is a development model of the Theory of Reasoned Action (TRA). In TAM, there are two factors. The main factors that affect the acceptance of technology are usability and convenience. This small number of constructs makes TAM a simple method that can rapidly measure the acceptance of new technology [25].

There are six constructs or variables in TAM, which are divided into core variables of user motivation (perceived usefulness, perceived ease of use, and attitudes toward technology on technology), external variables, and outcome variables (behavioral intention to use/intention of behavior and actual system use/use of technology). Of the six existing variables, perceived usefulness and ease of use are the most important variables that directly and indirectly affect the results [26].

The perceived usefulness variable is the degree to which a person believes an existing technology or system can improve performance [27]. People tend to use or not use mobile applications to the extent that they think that the application can help them do a better job. The variable perceived ease of use is the level of someone who believes that using technology or a system does not require as little effort as possible. If someone feels an application is helpful but difficult to use, and the difficulty is not worth the usefulness, this will affect the application’s use.

**Figure 1:**
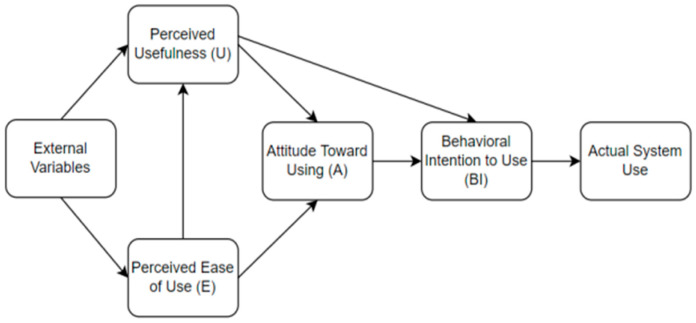
TAM Model.

In addition to the two main variables above, TAM is often accompanied by external factors that explain variations in ease of use and perceived usefulness [26]. Some of the most widely used external variables in the research are self-efficacy, subjective norm, enjoyment, computer anxiety, and experience [28]. The TAM model can be seen in Figure 1 below.

**Figure 2:**
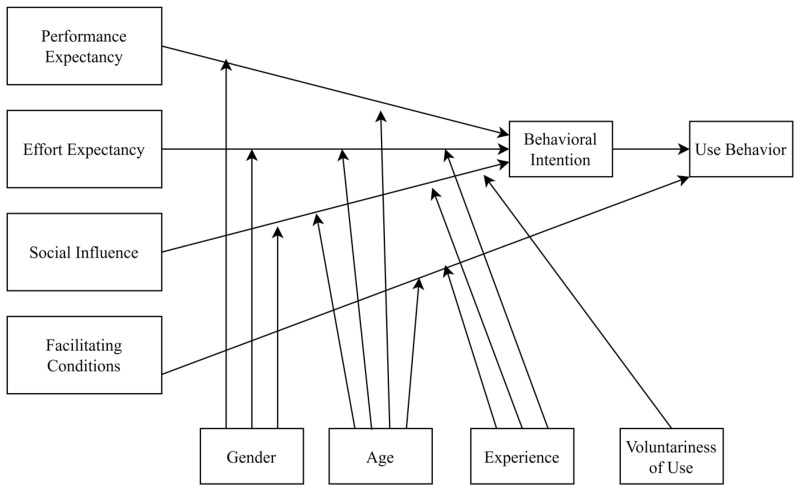
UTAUT Model (Venkatesh, Morris, Davis, and Davis, 2003).

The UTAUT model has been vastly used to evaluate technology acceptance [29]. UTAUT has four main constructs to explain technology acceptance [30]: performance expectancy, which measures the benefits derived from the usage of the studied technology; effort expectance, which captures the ease or difficulty related to the usage of the technology; social influence, which captures how usage of a technology by social influence might affect the user acceptance [31]; and facilitating conditions, which evaluate the level of technical infrastructure that supports and eases the usage of the technology [32]. The UTAUT model has also been used to evaluate such technologies such as recommenders [33], and chatbots [34]. The figure of the UTAUT model is shown in Figure 2 [30].

**Figure 3:**
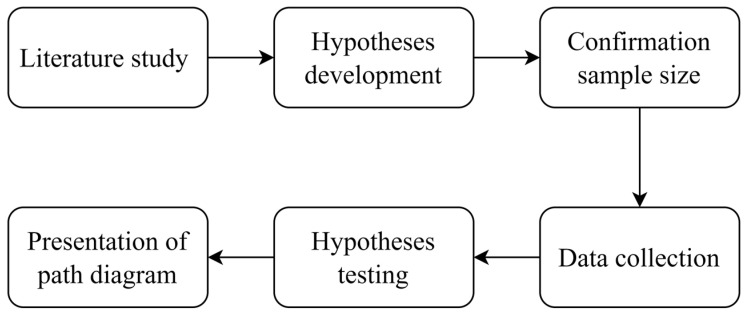
Research Methodology.

The methodology of this study has five primary phases which are shown in Figure 3. This approach is carried out with the aim of knowing whether digital applications for women are able to bridge the gap in increasing knowledge about financial literacy. With the implementation of digital applications in financial literacy education, women are becoming more aware of how they can manage finances, which is especially important for womenpreneurs, (those women who act as entrepreneurs and housewives at the same time).

### 2.1. Literature Study

The first phase of this study will review prior studies examining technology acceptance models from academic literature published within the last five years. Based on the previous studies, the use of UTAUT, TAM, or Usability was found to be the most common models used to measure the user’s technology acceptance.

### 2.2. Hypotheses Development

The hypotheses of this study are adopted based on UTAUT, TAM, and Usability model. There are six primary constructs used in this model with the following details, which can be seen in Table 1 [35,36,37]:

- Performance and Effort Expectancy: refers to the perception that technology can help the MSMEs’ owners to increase their business performance and measure how easy the system is to use.
- Facilitating Conditions: defined as a belief of the individuals that an organizational and technical infrastructure can support the use of the technology.
- Satisfaction: refers to how the users’ overall impression can affect their acceptance of the technology.
- Social Influence: evaluates how the usage of technology by relatives and acquaintances affect the user’s acceptance of it.
- Learning Attitude: refers to something that drives users’ intention to learn using technology.
- User Perception: degree of understanding of the concept of technology.
- Learning Intention: describes the commitment of users to learning using technology in the future.
- Continuance Intention: can be defined as an individual’s subjective probability that they would engage with the technology.

**Figure 4:**
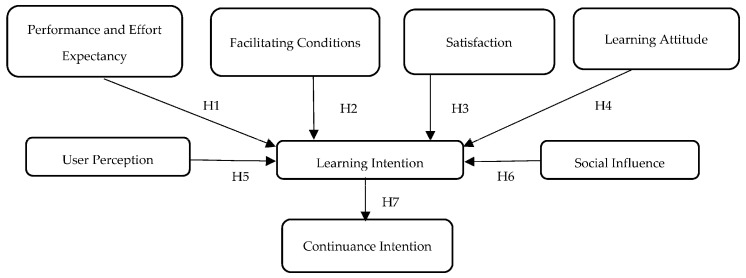
Theoretical research framework.

The theoretical research framework of this study is shown in Figure 4.

This study will test seven primary hypotheses:

**Hypotheses 1 (H1).**  *Performance and Effort Expectancy have a positive impact on Learning Intention*.

**Hypotheses 2 (H2).**  *Facilitating Conditions have a positive impact on Learning Intention*.

**Hypotheses 3 (H3).**  *Satisfaction has a positive impact on Learning Intention*.

**Hypotheses 4 (H4).**  *Learning Attitude has a positive impact on Learning Intention*.

**Hypotheses 5 (H5).**  *User Perception has a positive impact on Learning Intention*.

**Hypotheses 6 (H6).**  *Social Influence has a positive impact on Learning Intention*.

**Hypotheses 7 (H7).**  *Learning Intention has a positive impact on Continuance Intention*.

### 2.3. Confirmation Sample Size

The data for this research were collected from the Jabodetabek (Jakarta, Bogor, Depok, Tangerang, Bekasi) participants from Indonesia. The participants were from the group of womenpreneurs in the selected area. The sample size was determined based on the following Slovin formula.

(1) $$n=\frac{N}{1+Ne^{2}}$$

Whereas:

*n* = the size of the sample

*N* = population

*e* = margin of error.

All the participants will be tested with the designed prototype of the financial literacy e-learning application as shown in Appendix A, Table A3. 

### 2.4. Data Collection

The data was collected by using a questionnaire as a supportive tool to gather respondents’ opinions (See Appendix A: Table A1 and Table A2). There were 2 questionnaires that were used in this research: a user’s demographic questionnaire and a primary hypotheses questionnaire.

The user’s demographic questionnaire was focused on discovering the demographics of users, such as age, mobile phone ownership, and technology capability of the 130 respondents who filled out the questionnaire. The primary hypotheses questionnaire was constructed based on the primary hypotheses that was already conducted in the previous stage with 195 respondents. 

In addition, the respondents were womenpreneurs from Jakarta, Bogor, Depok, Tangerang, and Bekasi (Jabodetabek) City, Indonesia. The total number of respondents who filled the questionnaire are 130 people obtained from the user’s demographic questionnaire and 195 people from the primary hypotheses questionnaire, as determined by the sample number in using TAM methods.

### 2.5. Hypotheses Testing

The collected data will be calculated using the SmartPLS tool. Validity measurement will be conducted to determine the validity of the variables in every construct by using the Pearson product-moment with the following formula:

(2) $$r=\frac{n(\sum XY)-(\sum x)(\sum y)}{\sqrt{\left\{n\sum x^{2}-{\left(\sum x\right)}^{2}\}\{n\sum Y^{2}-{\left(\sum Y\right)}^{2}\right\}}}$$

The validity of every variable will be determined based on the result of the correlation coefficient variable (*r*) compared with the critical value. If the *r*-value > the critical value, then the variable will be considered valid. Furthermore, the critical value can be obtained by using the following formula:

(3) $$r_{CriticalValue}=\frac{t}{\sqrt{df+t^{2}}}$$

The reliability measurement was also conducted to determine the reliability of every variable used. The reliability testing will be shown with the following Cronbach’s alpha formula:

(4) $$\propto =\left(\frac{M}{M-1}\right)\left(1-\frac{\sum s_{t}}{s_{t}^{2}}\right)$$

The reliable questionnaire is shown with the value of the Cronbach’s alpha formula over 0.70 (Hair, Hult, Ringle and Sarstedt, 2017) [46]. On the other hand, the six hypotheses will be tested by measuring the path coefficient and the T-statistic. The path coefficient value describes the relationship path between one variable and another, and it has a range value between −1 and 1. Meanwhile, the T-statistic is used to determine if there is a significant difference between the means of two groups, which may be related to certain features. If the t-statistic value is >1.65, there is a significant relationship between those two groups. However, if the value is <1.65, there is no significant relationship between the groups.

The following questions shown in Table 2 for measuring the technology acceptance is based on the model above. The answer scale is from 1 (extremely disagree) to 5 (extremely agree).

## 3. Results

### 3.1. Respondence Profiles

#### 3.1.1. Age Generation of Womenpreneurs

**Figure 5:**
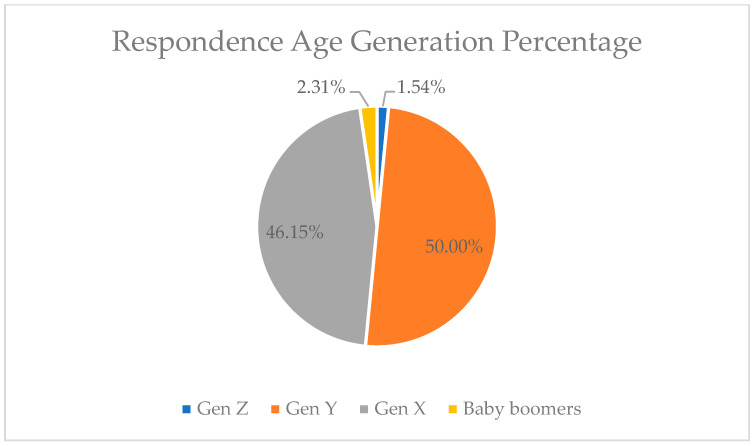
The Percentage of age generation.

Several factors of the usage of Information and Communication Technology (ICT) greatly influence its utilization, such as the age factor [47]. There are four age-generational groups that are currently used to generalize age groups, including the baby boomer generation, generation X, generation Y, and generation Z. The four generations have different age ranges, baby boomers are the oldest age, and generation Z is the youngest [48]. Based on the results of this research survey, the dominant user that is a highly considerable age generation is generation Y, and 50% of womenpreneurs have come from this generation. Therefore, in defining the acceptance of the technology, Gen Y have the highest acceptance rate, while baby boomers have the lowest rate. This can be seen in Table 2 and Figure 5. The developer must prioritize the needs of Gen Y, since the majority of the targeted users of digital financial literacy applications are from Gen Y. 

#### 3.1.2. Gadget Ownership of the Womenpreneurs

Smartphone ownership is assessed in this study. The aim is to see the ease with which the womenpreneurs can access the technology. The lack of ability to access the technology is affected by the challenges associated with smartphone ownership. Based on the results of the survey, it was found that smartphones owned by womenpreneurs were privately owned. This shows that womenpreneurs have high freedom in accessing information through technology (smartphones), due to the absence of the usage of the shared smartphone. This parameter was used to analyze the ease with which the intended users can access the prototype financial literacy e-learning smartphone application. For those without a solely owned smartphone, the opportunities to gain financial literacy knowledge will be more limited than for those womenpreneurs with their smartphone. This can be seen in Table 3.

#### 3.1.3. Technological Savvy of Womenpreneurs

**Figure 6:**
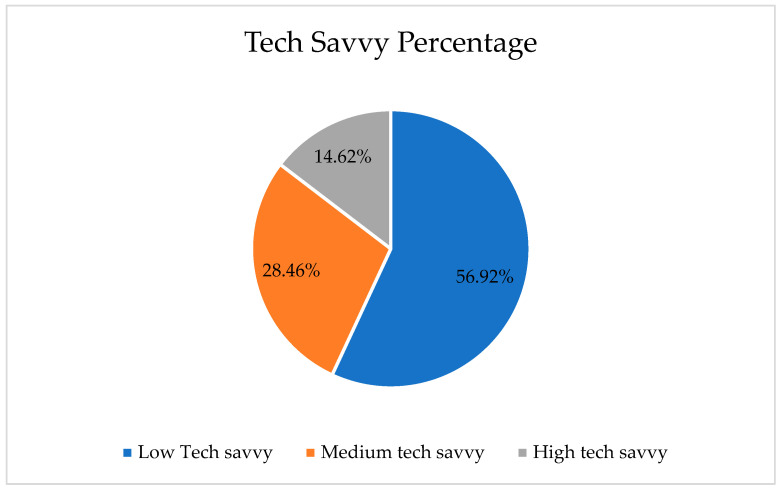
The percentage of technology savvy level.

The technological savvy of the intended users is one of the considerations in developing an application. Technological savvy for womenpreneurs was assessed based on their ability to utilize existing applications on their smartphones. In this study, womenpreneurs were asked how often they use applications in their daily life, and the number of different applications they use. This is due to their independency in accessing reliable information and communication regarding the use of existing applications. Based on this research, most of the womenpreneurs surveyed had low technological savvy levels, which in turn affects the capability of the intended users to adopt this particular application. The level of technological savvy among womenpreneurs can be seen in Table 4 and Figure 6. Hence, the design of the application must be easy to use and access, since the target users are not good at using smartphone applications. They are still struggling in using the simple smartphone application even it is a text applications. Therefore, the financial literacy apps must be well designed due to their low levels of technological savvy in addition to their lack of financial literacy education.

### 3.2. Validity and Reliability of the Questionnaire

The preliminary test was performed to confirm the validity and reliability of questionnaire. Kendall’s Tau-b test [35,41] was used to confirm the validity of questionnaire and Cronbach’s alpha formula was used to confirm reliability. As shown in Table 5 and Table 6, the validity and reliability tests on the questionnaire are confirmed to be appropriate. 

Table 5 shows that all of the indicators for the questionnaire have values of <0.05, which means all of the indicators in the questionnaire are valid. 

Table 6 shows that all of the variables in the questionnaire have an ∝> 0.7 [41]. This means all of the variables in the questionnaire are being reliably analyzed. After the preliminary test, a confirmatory factor analysis was performed to confirm the validity and reliability of the data. For validity, convergent validity and discriminant validity were used to confirm the validity of the data, and the composite reliability and the Cronbach alpha formula were used to confirm the reliability of the data. 

### 3.3. Convergent Validity

The loading factor and average variance extracted (AVE) were used to confirm the validity of the data. As shown in Table 7 and Table 8, three of the variables in the loading factor indicator were not confirmed to be appropriate and the AVE was confirmed to be appropriate.

Based on Table 7, all of the CI, LA, LI, S, and UP variables have a strong relationship and strongly influence their construct. As for FC and SI constructs, there’s one indicator each (FC3 and SI3) that has a weak relationship with their construct. While for PE construct, half of the indicators (PE3, PE5, and PE6) have a weak relationship with the PE construct and only weakly influence their construct. The bold number in Table 7 indicate that the factor has a weak relationship with the construct, due the number is under 0.7.

Based on Table 8, all of the variables have over >0.5 for AVE, which mean all of the indicators represent their construct. 

### 3.4. Discriminant Validity

Fornell Larcher criterion and cross-loading were used to confirm the validity of the data. As shown in Table 9 and Table 10, the discriminant validity was confirmed as appropriate.

Based on Table 9, all of the variables have a greater correlation value to themselves than the correlation value to other variables, which means all of the variables can be used in the research.

Based on Table 10, all of the variables have a greater correlation to their indicators than their correlation values with other indicators, which means all of the indicators can be used in the research

### 3.5. Reliability Test 

Composite reliability and Cronbach’s alpha were used to confirm the reliability of the data. As shown in Table 11 and Table 12, only one variable (SI) is not appropriate.

Based on Table 11 and Table 12, all the constructs, excluding the SI construct have good reliability as all of the values are >0.7. 

### 3.6. Evaluation of the Structural Equation Modeling (SEM)

**Figure 7:**
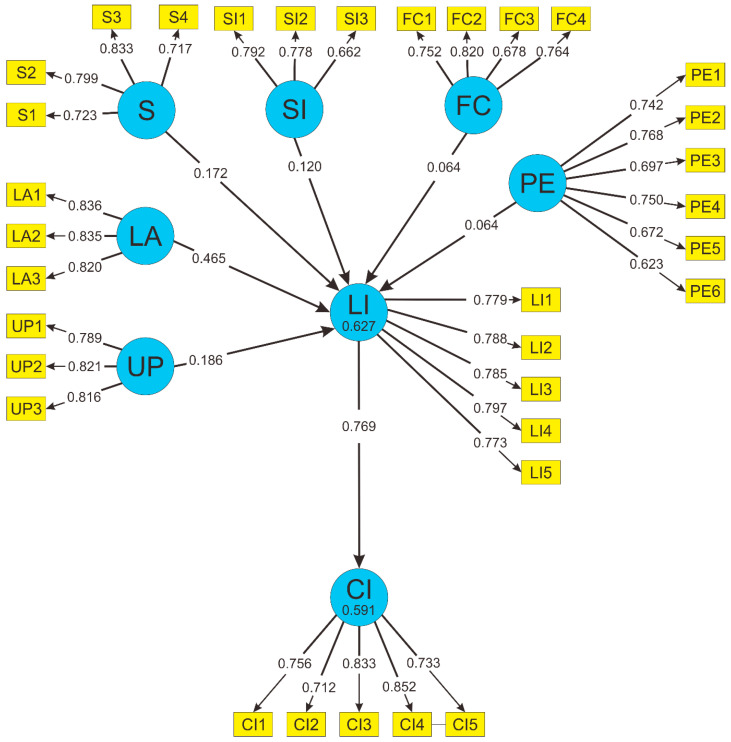
SEM with indicators of factors influencing womenpreneurs using mobile application.

Figure 7 demonstrates the Structural Equation Modeling (SEM) used for evaluating the factors influencing womenpreneurs using mobile applications. The evaluation of the SEM has five steps to confirm the model and the hypotheses.

#### 3.6.1. R Squared Analysis

R Squared analysis was used to assess the influence all of the variables have on the endogenous variables (CI and LI). As shown in Table 13, the variable CI has an R-Square value of 0.591 which means all of the variables in this research only influence the variable CI by 59.1%. The variable LI has an R-Square value of 0.627 which means all of the variables in this research only influence variable LI by 62.7%.

#### 3.6.2. Model Fit

Model fit was used to confirm whether the model was fit or not. As shown in Table 14, the model has a value of 0.638 which means it is fit and can be used in the research.

#### 3.6.3. Predictive Relevance

Predictive relevance was used to confirm all of the variables based on hypotheses that show good observation. As shown in Table 15, CI has 0.487 and LI has 0.576 for Q2 which means all of the endogenous variables have a good observation. 

#### 3.6.4. Path Coefficient

The path coefficient was used to examine the correlation between exogenous and endogenous variables. As shown in Table 16, all of the variables have a positive correlation, excluding the exogenous variable FC which has a negative correlation to the endogenous variable LI.

#### 3.6.5. T-Statistic

The T-Statistic was used to confirm whether the hypothesis was accepted or not. As shown in Table 17, the LA → LI, LI → CI, S → LI, and UP → LI paths all have a positive and significant relationship. However, even though the PE → LI and SI → LI paths have positive relationships, they are both insignificant relationships, and these hypotheses are to be rejected. 

## 4. Discussion

Based on UTAUT, TAM, and Usability theory, this study explores the factors which influence the technology acceptance of lower-middle socio-economic users, especially in financial literacy education. It is worth noting that the variables of facilitating conditions, performance and effort expectancy, and social influence have no significant effect on learning intention.

Firstly, through verification of the Technology Acceptance Model, we found that learning attitude (T = 6.166, *p* = 0.000) has a significant effect on lower-middle socio-economic users’ learning intentions (H4 accepted). In this study, among the three observed variables which represent learning attitude, LA3 “*I am interested to know more about financial literacy through this app*” has the highest loading factors. It can be seen that when users want to know more about financial literacy through this app, their learning intention is higher. This finding is similar to the conclusion drawn by Peng et al. [37] which indicates that some internal motivation drives users’ intentions to gain financial literacy knowledge through the app. 

Furthermore, satisfaction (T = 2.016, *p* = 0.044) and user perception (T = 2.210, *p* = 0.027) also have a significant effect on lower-middle socio-economic users’ learning intentions (H3 and H5 accepted). If the e-learning platform has all the features needed to enhance their financial literacy knowledge (S3), it would have a higher impact on users’ learning intentions since the observed variables have the highest loading factor. Research by Zhou et al. [41] shows that satisfaction has a significant effect on app usability which influences learning intention. For example, when the app has all the features needed to meet their needs or improve their knowledge, it would have a higher impact on users’ learning intentions. The higher the learning intention is, the greater the continuance intention as well (T = 22.867, *p* = 0.000). Since learning intention has a significant effect on continuance intention, this means all the variables which have a significant effect on learning intention will also affect continuance intention (H7 accepted). Users who say they will frequently use the app in the future to broaden their financial literacy knowledge (CI4) have the highest loading factors among all the observed variables. For example, when users have a higher learning intention, they would frequently use the app since it benefits them. Equally true is that when users realize that the app is working well for them, it also enhances their intentions to learn the app (Sukendro et al. [18]; Listyani [7]; Lu et al. [44]). This means user perception also has an important role in learning intention. The higher the understanding of users on how important this app is in improving their business, the higher the intention to learn the app (Wang, L.Y.K. et al. [36]). However, there are three factors from technology acceptance theory that did not have a significant effect on facilitating conditions: social influence, and performance and effort expectancy. 

Unlike the conclusion by Curtale et al. [40], in this study, facilitating conditions (T = 0.9, *p* = 0.368) had no significant effect on learning intention in this research (H2 rejected). This happens because a lack of sufficient resources and limited support can prevent individuals from accepting the app [27]. According to Venkatesh et al. [30], facilitating conditions do not affect learning intention. Moreover, one of the observed variables states that: “The mobile internet used to access the e-learning platform is still affordable” (FC3) has a weak relationship to its variable. This factor is the main reason why users have difficulty accessing the e-learning app. The next factor, social influence (T = 1.840, *p* = 0.066) did not have a significant effect on learning intention (H6 rejected). Unlike the conclusion by Abrahão et al. [42], the findings of Venkatesh et al. [30] from the multiple path analysis showed that social influence did not affect learning intention. This happens because one of the observed variables which states “*people who are important to me could assist me in the use of an e-learning platform to master my financial literacy skills*” (SI3) has a weak relation to its variable. Wang et al. [36] state that people may simply not know if they will like the app before they use it, which means people should have first-hand use experience before data is collected. For example, when people already have experience using the app, these people can then influence or teach other people to use the app too. Venkatesh et al. [30] also state the app’s performance and the social environment it is used in may influence people’s intention to use the app for the first time. The last variable that has no significant effect on learning intention is performance expectancy (T = 0.938, *p* = 0.348) (H1 rejected). Unlike the conclusion by Jung et al. [38] and Raffaghelli et al. [39], the findings of Venkatesh et al. [30] also state that performance expectancy did not affect learning intention. This happens because among six of the observed variables, three of them have weak relationships to their variable (PE3, PE5, and PE6). PE3 states the expectation that learning financial literacy can be helpful; PE5 states that learning to navigate within the app was easy due to the transparent icon; and PE6 states it was easy to become skillful at using the app, so the system can support improving financial literacy. 

It was seen in this study that the intended users (womenpreneurs) chose not to adopt the technology due to an inability to perceive of the app’s benefits. Even though the womenpreneurs had great expectations for the financial literacy app as a digital platform, the difficulty many encountered trying to learn to use it, which can be a driving factor contributing to their failure to benefit from using this app. Because many lower-middle socio-economic users (including most womenpreneurs) lack sufficient understanding of technology to properly use this app, this results in an absence of significant statistical relationships between their performance expectancy, social influence, and facilitating condition which can help them improve their financial literacy knowledge using a digital platform.

The implementation of a digital platform into a financial literacy education program will not help womenpreneurs improve their financial literacy knowledge. It would actually have the opposite result. Because a digital platform was found to increase the difficulty in learning financial literacy, the use a of digital platforms would lead many womenpreneurs to give up entirely on learning financial literacy. This shows that a digital platform is not the right tool to teach financial literacy to womenpreneurs in Indonesia. 

## 5. Conclusions

Firstly, by utilizing the Technology Acceptance Model, we found that the learning attitude variable (T = 6.166, *p* = 0.000) has a significant effect on lower-middle socio-economic users’ learning intentions (H4 accepted). In this study, among the three observed variables which represent learning attitude, LA3 “*I am interested to know more about financial literacy through this app*” has the highest loading factors. It has been observed that when users want to know more about financial literacy through this app, their learning intentions are higher. Furthermore, satisfaction (T = 2.016, *p* = 0.044) and user perception (T = 2.210, *p* = 0.027) also have a significant effect on lower-middle socio-economic users’ learning intentions (H3 and H5 accepted). Additionally, because the learning intention variable was seen to have a significant effect on the continuance intention variable, this implies that if all the variables have a significant effect on learning intention, then all variables would also have an impact on continuance intention (H7 accepted). The next factor, social influence (T = 1.840, *p* = 0.066), did not have a significant effect on learning intention (H6 rejected). In this study, facilitating conditions (T = 0.9, *p* = 0.368) were found to have no significant effect on learning intention (H2 rejected). The final variable, performance expectancy (T = 0.938, *p* = 0.348) also had no significant effect on learning intention (H1 rejected). This study found that womenpreneurs did not want to adopt the technology because they did not understand the app’s benefits. Therefore, the development of a digital platform to offer financial literacy education is not the appropriate method to support the womenpreneurs in Indonesia to improve their financial literacy knowledge. This shows that womenpreneurs in Indonesia would benefit more from non-digital methods of providing financial literacy education.

## 6. Limitations and Future Research

This study mainly focused on investigating the factors that influence womenpreneurs’ intentions to use technology. The study results may have important implications and is believed to be helpful for the development of mobile applications for womenpreneurs. Although this study has interesting results, it is necessary to consider its limitations due to its limited demographics. The researchers successfully carried out the study within the scope of its objectives, the potential remains for further research to be conducted by including more variables from different theories and models, and by considering additional social issues. Moreover, the sample size can be broadened to analyze a larger portion of the Indonesian population. Additionally, a macro level enterprise also can be examined as a subject for future research. Further studies on non-technology-based methods (such as face-to-face teaching) to support womenpreneurs in Indonesia to improve their financial literacy knowledge are also recommended. 

## Figures and Tables

**Table 1:** The construct and measurement items.

Construct	Items	Measures	Supporting Measures
Performance Expectancy (PE)	PE1	I believe learning financial literacy through the e-learning platform can be useful in my daily life.	[38,39]
	PE2	I believe using the e-learning platform can help me understand financial literacy quickly.	
	PE3	I believe using all the financial literacy learning material in the e-learning platform can be helpful for my MSME productivity.	
	PE4	I found it easy to use the e-learning prototype to help me gain a better understanding of financial literacy.	
	PE5	I think that learning the navigation of the e-learning prototype was easy for me due to the clear icon.	
	PE6	I think that it was easy for me to become skillfull at using the e-learning prototype, so the system supported me in my financial literacy process.	
Facilitating Conditions (FC)	FCI	I have the basic knowledge of using technology in my daily life	[40]
	FC2	I have the resources such as mobile devices and the internet to support me in using technology.	
	FC3	The mobile internet used to access the e-learning platform is still affordable.	
	FC4	Overall, the usage of e-learning technology (internet and mobile device) is reasonably priced.	
Satisfaction (S)	SI	The information in the e-learning application is well-organized, so I could easily find the needed information.	[41]
	S2	The e-learning platform has all the information needed regarding financial literacy.	
	S3	The e-learning platform has all the features needed to enhance my financial literacy knowledge.	
	S4	Overall, I like the interface of the app. and I am satisfied with it.	
Social Influence (SI)	SII	People in my neighborhood would think I should use technology to learn about financial literacy nowadays.	[42]
	S12	People from my family think I should start using technology to support my MSME productivity.	
	S13	People who are important to me could assist me in the use of e-learning platforms to master my financial literacy skills.	
Learning Attitude (LA)	LAI	It is fun to learn financial literacy through the mobile app.	[37]
	LA2	It is worthwhile to have a knowledge of financial literacy.	
	LA3	I am interested to know more about financial literacy through this app.	
User Perception (UP)	UPI	I understand that the use of technology to gain financial literacy knowledge is inevitable.	[36]
	UP2	I understand that gaining more knowledge of financial literacy can help my MSME.	
	UP3	I understand learning with technology could be more flexible and effective.	
Learning Intention (LI)	LII	I am ready to do everything to gain financial literacy knowledge through this application.	[43]
	LI2	I will make every effort to participate in a financial literacy class in this application.	
	LI3	I have a very serious thoughts about actively participating in a financial literacy class in the future.	
	LI4	I am determined to broaden my financial literacy knowledge.	
	LI5	I have the learning intention to help my MSME grow.	
Continuance Intention (CI)	CII	I will use this e-learning platform in the future when the full version is released.	[18,44,45]
	CI2	I would recommend to my relatives to use the e-learning platform to enhance their financial literacy knowledge.	
	CI3	I believe using technology will benefit my business at one point.	
	CI4	I will frequently use the prototype in the future to broaden my financial literacy knowledge.	
	CI5	My intention to use the application in the future is great compared to using any alternative means.	

**Table 2:** Age generation of survey result.

Generation	Age	Number	Percentage
Gen Z	10–25 years old	2	1.54%
Gen Y	26–41 years old	65	50.00%
Gen X	42–57 years old	60	46.15%
Baby boomers	58–76 years old	3	2.31%
**Total**		130	100%

**Table 3:** The ownership of smartphones.

Smartphone Usage	Number	Percentage
Family-owned	12	9.23%
Sharing	13	10.00%
Self-owned	105	80.77%
**Total**	130	100.00%

**Table 4:** Technological savvy levels of womenpreneurs.

Tech Savvy Level	Number	Percentage
Low Tech savvy	74	56.92%
Medium tech savvy	37	28.46%
High tech savvy	19	14.62%
**Total**	130	100.00%

**Table 5:** Validity test results for the questionnaire.

Construct	Item	Validity
Performance Expectancy (PE)	PE1	Valid
	PE2	Valid
	PE3	Valid
	PE4	Valid
	PE5	Valid
	PE6	Valid
Facilitating Conditions (FC)	FC1	Valid
	FC2	Valid
	FC3	Valid
	FC4	Valid
Satisfaction (S)	S1	Valid
	S2	Valid
	S3	Valid
	S4	Valid
Social Influence (SI)	SI1	Valid
	SI2	Valid
	SI3	Valid
Learning Attitude (LA)	LA1	Valid
	LA2	Valid
	LA3	Valid
User Perception (UP)	UP1	Valid
	UP2	Valid
	UP3	Valid
Learning Intention (LI)	LI1	Valid
	LI2	Valid
	LI3	Valid
	LI4	Valid
	LI5	Valid
Continuance Intention (CI)	CI1	Valid
	CI2	Valid
	CI3	Valid
	CI4	Valid
	CI5	Valid

**Table 6:** Reliability test of the questionnaire.

Construct	Alpha	Reliability
Performance Expectancy (PE)	0.823	Reliable
Facilitating Conditions (FC)	0.753	Reliable
Satisfaction (S)	0.667	Reliable
Social Influence (SI)	0.787	Reliable
Learning Attitude (LA)	0.726	Reliable
User Perception (UP)	0.803	Reliable
Learning Intention (LI)	0.885	Reliable
Continuance Intention (CI)	0.877	Reliable

**Table 7:** Loading Factor.

	CI	FC	LA	LI	PE	S	SI	UP
**CI1**	0.756							
**CI2**	0.712							
**CI3**	0.833							
**CI4**	0.853							
**CI5**	0.733							
**FC1**		0.752						
**FC2**		0.82						
**FC3**		**0.678**						
**FC4**		0.764						
**LA1**			0.836					
**LA2**			0.835					
**LA3**			0.850					
**LI1**				0.779				
**LI2**				0.788				
**LI3**				0.785				
**LI4**				0.797				
**LI5**				0.773				
**PE1**					0.742			
**PE2**					0.768			
**PE3**					**0.697**			
**PE4**					0.75			
**PE5**					**0.672**			
**PE6**					**0.623**			
**S1**						0.723		
**S2**						0.799		
**S3**						0.833		
**S4**						0.717		
**SI1**							0.792	
**SI2**							0.778	
**SI3**							**0.662**	
**UP1**								0.789
**UP2**								0.821
**UP3**								0.816

The bold number indicates that the number is <0.7.

**Table 8:** Average Variance Extracted.

	Average Variance Extracted (AVE)
CI	**0.607**
FC	**0.570**
LA	**0.706**
LI	**0.615**
PE	**0.505**
S	**0.592**
SI	**0.557**
UP	**0.654**

The bold number indicates that the number is >0.5.

**Table 9:** Fornell Larcher Criterion.

	CI	FC	LA	LI	PE	S	SI	UP
CI	**0.779**							
FC	0.489	**0.755**						
LA	0.647	0.491	**0.84**					
LI	0.769	0.486	0.733	**0.784**				
PE	0.53	0.619	0.503	0.53	**0.71**			
S	0.575	0.637	0.57	0.613	0.622	**0.769**		
SI	0.537	0.566	0.46	0.506	0.545	0.513	**0.746**	
UP	0.566	0.56	0.611	0.63	0.535	0.619	0.46	**0.809**

The bold number indicates that the number is >0.7.

**Table 10:** Cross Loading.

	CI	FC	LA	LI	PE	S	SI	UP
CI1	**0.756**	0.344	0.491	0.557	0.357	0.431	0.374	0.345
CI2	**0.712**	0.399	0.48	0.565	0.24	0.318	0.36	0.531
CI3	**0.833**	0.368	0.53	0.656	0.472	0.548	0.461	0.462
CI4	**0.853**	0.397	0.545	0.679	0.534	0.493	0.495	0.44
CI5	**0.733**	0.404	0.47	0.52	0.438	0.434	0.387	0.435
FC1	0.379	**0.752**	0.36	0.385	0.505	0.471	0.362	0.387
FC2	0.41	**0.82**	0.425	0.385	0.513	0.48	0.445	0.478
FC3	0.297	**0.678**	0.321	0.304	0.378	0.374	0.421	0.433
FC4	0.378	**0.764**	0.371	0.384	0.461	0.582	0.485	0.403
LA1	0.572	0.459	**0.836**	0.618	0.486	0.48	0.4	0.573
LA2	0.529	0.437	**0.835**	0.61	0.413	0.531	0.43	0.518
LA3	0.528	0.341	**0.85**	0.618	0.369	0.426	0.329	0.448
LI1	0.612	0.42	0.568	**0.779**	0.449	0.452	0.41	0.485
LI2	0.565	0.48	0.567	**0.788**	0.419	0.501	0.458	0.554
LI3	0.597	0.329	0.557	**0.785**	0.351	0.442	0.359	0.501
LI4	0.597	0.366	0.554	**0.797**	0.387	0.458	0.335	0.476
LI5	0.64	0.314	0.622	**0.773**	0.467	0.545	0.42	0.457
PE1	0.447	0.433	0.378	0.449	**0.742**	0.512	0.389	0.41
PE2	0.428	0.505	0.462	0.431	**0.768**	0.476	0.397	0.415
PE3	0.342	0.471	0.357	0.34	**0.697**	0.435	0.398	0.465
PE4	0.351	0.471	0.358	0.411	**0.75**	0.448	0.413	0.31
PE5	0.29	0.365	0.21	0.267	**0.672**	0.327	0.355	0.288
PE6	0.371	0.375	0.331	0.307	**0.623**	0.417	0.374	0.393
S1	0.416	0.439	0.44	0.363	0.442	**0.723**	0.369	0.439
S2	0.42	0.568	0.474	0.508	0.511	**0.799**	0.464	0.513
S3	0.428	0.473	0.443	0.542	0.472	**0.833**	0.365	0.515
S4	0.519	0.475	0.403	0.445	0.49	**0.717**	0.384	0.429
SI1	0.355	0.449	0.277	0.341	0.367	0.339	**0.792**	0.395
SI2	0.487	0.456	0.412	0.461	0.448	0.493	**0.778**	0.336
SI3	0.33	0.348	0.321	0.302	0.395	0.273	**0.662**	0.302
UP1	0.441	0.396	0.472	0.509	0.393	0.475	0.348	**0.789**
UP2	0.416	0.501	0.535	0.486	0.423	0.462	0.398	**0.821**
UP3	0.513	0.464	0.475	0.531	0.48	0.56	0.372	**0.816**

The bold number indicates that the number is >0.6.

**Table 11:** Composite Reliability.

	Composite Reliability
CI	0.847
FC	0.755
LA	0.792
LI	0.844
PE	0.817
S	0.785
SI	0.626
UP	0.736

**Table 12:** Cronbach’s Alpha.

	Cronbach’s Alpha
CI	0.837
FC	0.748
LA	0.792
LI	0.844
PE	0.805
S	0.77
SI	0.612
UP	0.735

**Table 13:** R Square.

	**R Square**
CI	0.591
LI	0.627

**Table 14:** Model Fit.

	**Model Fit**
NFI	0.638

**Table 15:** Predictive Relevance.

	Predictive Relevance
CI	0.487
LI	0.576

**Table 16:** Path Coefficient.

	CI	FC	LA	LI	PE	S	SI	UP
CI								
FC				−0.064				
LA				0.465				
LI	0.769							
PE				0.064				
S				0.172				
SI				0.12				
UP				0.186				

**Table 17:** T-Statistics.

	T-Statistic	*p*-Value	Hypothesis
FC → LI	0.9	0.368	Rejected
LA → LI	6.166	0.000	Accepted
LI → CI	22.867	0.000	Accepted
PE → LI	0.938	0.348	Rejected
S → LI	2.016	0.044	Accepted
SI → LI	1.84	0.066	Rejected
UP → LI	2.21	0.027	Accepted

## Appendix A

**Table A1:** User’s demographic questionnaire of survey.

**Name:**	
**Age:**	
**Gender:**	
**Phone ownership:**	
**Experience in using technology (year):**	
**The used application in smartphone:**	

**Table A2:** Primary hypotheses questionnaire of survey.

Name:						
Age:						
No	Questions	ED	D	N	A	EA
**Performance Expectancy** [38,39]						
1	I believe learning financial literacy through the e-learning platform can be helpful in my daily life.					
2	I believe using the e-learning platform can help me understand financial literacy quickly.					
3	I believe using all the financial literacy learning material in the e-learning platform can be helpful for my MSME productivity.					
4	I found it easy to use the e-learning prototype to help me gain a better understanding of Financial literacy.					
5	I think that learning the navigation of the e-learning prototype was easy for me due to the clear icon.					
6	I think that it was easy for me to become skilful at using the e-learning prototype, so the system supported me in my financial literacy process.					
**Facilitating Conditions** [40]						
1	I have the basic knowledge of using technology in my daily life.					
2	I have the resources such as mobile devices and the internet to support me in using technology.					
3	The mobile internet used to access the e-learning platform is still affordable.					
4	Overall, the usage of e-learning technology (internet and mobile device) are reasonably priced.					
**Satisfaction** [41]						
1	The information in the e-learning application is well-organized, so I could easily find the needed information.					
2	The e-learning platform has all the information needed regarding financial literacy.					
3	The e-learning platform has all the features needed to enhance my financial literacy knowledge.					
4	Overall, I like the interface of the app, and I am satisfied with it.					
**Social Influence** [42]						
1	People in my neighbourhood would think I should use technology to learn about financial literacy nowadays.					
2	People from my family think I should start using technology to support my MSME productivity.					
3	People who are important to me could assist me in the use of e-learning platforms to master my financial literacy skills.					
**Learning Attitude** [37]						
1	It is fun to learn financial literacy through the mobile app.					
2	It is worthwhile to have a knowledge of financial literacy.					
3	I am interested to know more about financial literacy through this app.					
**User Perception** [36]						
1	I understand that the use of technology to gain financial literacy knowledge is inevitable.					
2	I understand that gaining more knowledge of financial literacy can help my MSME.					
3	I understand learning with technology could be more flexible and effective.					
**Learning Intention** [43]						
1	I am ready to do everything to gain financial literacy knowledge through this application.					
2	I will make every effort to participate in a financial literacy class in this application.					
3	I have a very serious thought about actively participating in a financial literacy class in the future.					
4	I am determined to broaden my financial literacy knowledge.					
5	I have the learning intention to help my MSME grow.					
**Continuance Intention** [18,45,48]						
1	I will use this e-learning platform in the future when the full version is released.					
2	I would recommend to my relatives to use the e-learning platform to enhance their financial literacy knowledge.					
3	I believe using technology will benefit my business at one point.					
4	I will frequently use the prototype in the future to broaden my financial literacy knowledge.					
5	My intention to use the application in the future is great compared to using any alternative means.					

**Table A3:** List of task.

No	Task List	Expected Output
1	Please access the learning materials through the e-learning prototype	
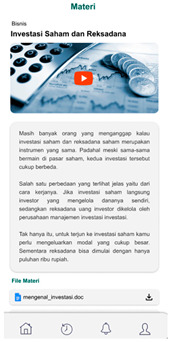
2	Please access the assignment page through the e-learning prototype	
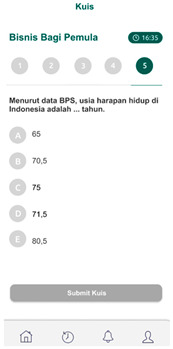
3	Please access the forum page and post a new feed through the e-learning prototype	
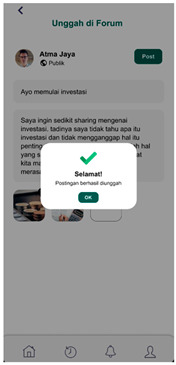
4	Please access the community group through the e-learning prototype	
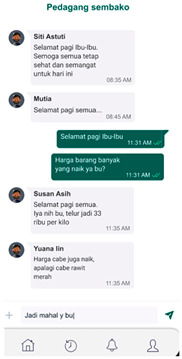
5	Please access the Q&A page through the e-learning prototype	
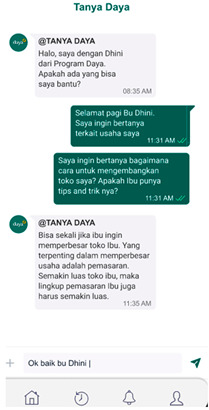
6	Please access the notification feature of the e-learning prototype	
